# PET Radiopharmaceuticals for Imaging Integrin Expression: Tracers in Clinical Studies and Recent Developments

**DOI:** 10.1155/2014/871609

**Published:** 2014-06-11

**Authors:** Roland Haubner, Simone Maschauer, Olaf Prante

**Affiliations:** ^1^Department of Nuclear Medicine, Innsbruck Medical University, Anichstraße 35, 6020 Innsbruck, Austria; ^2^Molecular Imaging and Radiochemistry, Department of Nuclear Medicine, Friedrich-Alexander University, Schwabachanlage 6, 91054 Erlangen, Germany

## Abstract

Noninvasive determination of integrin expression has become an interesting approach in nuclear medicine. Since the discovery of the first ^18^F-labeled cyclic RGD peptide as radiotracer for imaging integrin *α*_*v*_ *β*_3_ expression in vivo, there have been carried out enormous efforts to develop RGD peptides for PET imaging. Moreover, in recent years, additional integrins, including *α*_5_ *β*_1_ and *α*_*v*_ *β*_6_, came into the focus of pharmaceutical radiochemistry. This review will discuss the tracers already evaluated in clinical trials and summarize the preliminary outcome. It will also give an overview on recent developments to further optimize the first-generation compounds such as [^18^F]Galacto-RGD. This includes recently developed ^18^F-labeling strategies and also new approaches in ^68^Ga-complex chemistry. Furthermore, the approaches to develop radiopharmaceuticals targeting integrin *α*_5_ *β*_1_ and *α*_*v*_ *β*_6_ will be summarized and discussed.

## 1. Introduction

Integrins are heterodimeric glycoproteins consisting of an *α*- and *β*-subunit. There are 24 different combinations of the eight *β*-units and the eighteen *α*-units known. The integrins mediate cell-cell and cell-matrix interactions and transduce signals across the plasma membrane via insight-out and outside-in signaling [1]. Some of the integrins play an important role during migration of endothelial as well as tumor cells during tumor-induced angiogenesis and tumor metastasis. Angiogenesis, the formation of new blood vessels out of the preexisting vasculature, is a critical step in the development and dissemination of many human tumors. A variety of therapeutic strategies in oncology are focused on the inhibition of tumor-induced angiogenesis [2–4]. This includes approaches to inhibit VEGF, MMP, or integrin interactions. Concerning the integrins, most attention has been paid to the role of integrin *α*_*v*_ *β*_3_ and *α*_*v*_ *β*_5_ as they are prominent on proliferating vascular endothelial cells [5].

Thus, one of the most prominent target structures used for the development of radiopharmaceuticals for imaging angiogenesis is the integrin *α*_*v*_ *β*_3_ [6]. It has been shown that this integrin is involved in endothelial cell/matrix interaction during tumor-induced formation of new vessels as well as in mediation of tumor cell migration during invasion and extravasation [7]. A series of studies using a variety of different radiopharmaceuticals have already demonstrated that noninvasive determination of *α*_*v*_ *β*_3_ expression is feasible (for review, see [6, 8]).

In contrast to the data found in a variety of inhibition studies, which suggest a critical role for *α*_*v*_ *β*_3_ in angiogenesis, genetic studies indicate that the integrin *α*_*v*_ *β*_3_ is not required for angiogenesis [5]. An explanation for this discrepancy could be findings that animals lacking *α*_*v*_ *β*_3_ develop compensatory changes in VEGF signaling, which permit angiogenesis to occur during embryogenesis [9]. Anyway, genetic ablation of the integrin *α*_5_ *β*_1_, the major fibronectin-binding integrin, leads to severe vascular abnormalities [10] indicating that this integrin may play an even more important role as the integrin *α*_*v*_ *β*_3_ in neovascularization. Additionally, this integrin is upregulated in tumor blood vessels and plays a role in tumor angiogenesis and tumor growth [11, 12]. Thus, recently this integrin became another target structure in the development of radiopharmaceuticals for imaging angiogenesis.

A third class of tracer developed for the noninvasive determination of integrin expression focus on the integrin *α*_*v*_ *β*_6_. This integrin is unique in that it is exclusively expressed on epithelial cells [13]. It is highly upregulated during development of lung, skin, and kidney epithelia but its expression is low in healthy adult epithelia [14]. Elevated expression in adults is found only during wound healing [15]. It is found to regulate epithelial remodeling during development and tissue repair. Thus, it became an interesting target in tracer development because integrin *α*_*v*_ *β*_6_ is also found to be highly expressed on a variety of tumors including carcinoma of the breast, lung, colon, stomach, and oral and skin squamous cell carcinoma [13] and is associated with a more aggressive disease outcome [16].

There are already a variety of reviews dealing with the development of tracer targeting the integrins *α*_*v*_ *β*_3_/*α*_*v*_ *β*_5_ [6, 8, 25–27]. On the one hand, this review will focus on compounds which are already in clinical studies and, on the other hand, highlight most recent aspects of the preclinical development of tracer targeting these integrins. Moreover, it will summarize the developments concerning radiopharmaceuticals targeting the integrins, *α*_5_ *β*_1_ and *α*_*v*_ *β*_6_, which came most recently in the focus for PET tracer development (Table 1).

## 2. Tracer Targeting Integrin *α*_*v*_ *β*_3_/*α*_*v*_ *β*_5_

### 2.1. Tracers Already in Clinical Studies

#### 2.1.1. [^18^F]Galacto-RGD

**Figure 1:**
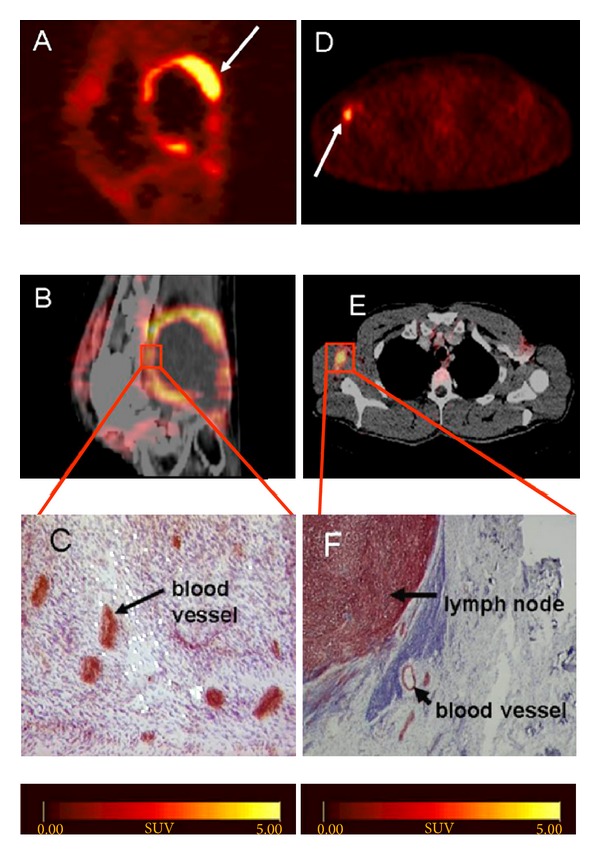
[^18^F]Galacto-RGD PET: (A–C) patient with a soft tissue sarcoma dorsal of the right knee joint. (A) Sagittal section acquired 170 min p.i. (B) PET/CT image fusion. (C) Immunohistochemistry of a peripheral tumor section using the anti-*α*_*v*_ *β*_3_ monoclonal antibody LM609 demonstrates intense staining predominantly of tumor vasculature. (D–F) Patient with malignant melanoma and a lymph node metastasis in the right axilla. (D) Axial section acquired 140 min p.i. (E) PET/Ct image fusion. (F) Immunohistochemistry of the lymph node demonstrates intense staining predominantly of tumor cells and also blood vessels (with permission from Haubner et al. [17]).

The first target structure used for the development of radiopharmaceuticals was the integrin *α*_*v*_ *β*_3_ [46]. Among the great variety of compounds introduced meanwhile, only a small set entered clinical studies. The first compound studied in patients was [^18^F]Galacto-RGD. This compound was developed based on an optimization strategy introducing sugar moieties to improve the pharmacokinetics [28, 47]. Initial clinical studies showed that the tracer was well tolerated with no severe side effects [48–50]. The effective dose calculated from an i.v. injection of [^18^F]Galacto-RGD was found to be approximately 0.02 mSv/MBq [50], being in the range of a routine [^18^F]FDG-PET scan [51]. The tracer was rapidly cleared predominately via kidneys, resulting in good tumor/background ratios. The highest background uptake was found in kidneys, liver, spleen, and intestine. Tumor uptake showed high variability and standard uptake values (SUV) ranged from 1.2 to 10. An additional study including 19 patients compared [^18^F]Galacto-RGD uptake in the lesions with immunohistochemical staining after tumor resection using angiogenesis markers (Figure 1) [17, 49].

A good correlation between tracer uptake and *α*_*v*_ *β*_3_ expression as well as microvessel density was found. In further investigations, the detection rate of a variety of different malignant lesions was studied including sarcoma, melanoma, renal cell cancer, squamous cell carcinoma of the head and neck, breast cancer, and glioblastoma multiforme [52–54]. In general, detection of the primary tumor was high (80%−100%) with a lower detection rate for lymph nodes and distant metastases. It has to be mentioned that the different studies also revealed that chronic inflammatory lesions like villonodular synovitis can also show significant uptake of [^18^F]Galacto-RGD [17], raising the same problem as with [^18^F]FDG that the tracer does not clearly differentiate between benign and malignant lesions. All the clinical as well as the preclinical data (which are not discussed here) have demonstrated that specific imaging of integrin *α*_*v*_ *β*_3_ expression is feasible using [^18^F]Galacto-RGD and PET; however, it has to be kept in mind that this receptor is not only expressed on endothelial cells during neovascularization but can also be present on the tumor cells themselves. Static PET imaging cannot distinguish the origin of the signal; thus, solely assessing angiogenesis is only possible if the tumor cells do not express the receptor.

Integrin *α*_*v*_ *β*_3_ is also expressed by macrophages and angiogenic endothelial cells in atherosclerotic lesions [55, 56]. Based on this, Beer et al. studied the potential of [^18^F]Galacto-RGD as a probe for imaging plaque inflammation and plaque vulnerability [57]. The pilot study including 10 patients with high-grade carotid artery stenosis scheduled for carotid endarterectomy revealed specific tracer accumulation in atherosclerotic carotid plagues and correlation of the tracer uptake with *α*_*v*_ *β*_3_ expression analyzed by immunohistochemical staining of the surgical specimen. Based on the promising initial results it was concluded that larger prospective studies have to be carried out to fully evaluate the potential of molecular imaging of integrin *α*_*v*_ *β*_3_ expression for the assessment of plaque inflammation in patients.

#### 2.1.2. [^18^F]Fluciclatide

Another integrin *α*_*v*_ *β*_3_/*α*_*v*_ *β*_5_ targeting PET radiopharmaceutical, which has already been studied in patients, is [^18^F]Fluciclatide. Similar to [^18^F]Galacto-RGD, this peptide derivative includes the RGD sequence as binding motif, but in contrast to the backbone cyclization found in [^18^F]Galacto-RGD this compound is cyclized via a thioether and a disulfide bridge. As a pharmacokinetic modifier, polyethylene glycol (PEG), instead of the sugar moiety and for radiolabeling an aminooxy function, was introduced. The labeling with ^18^F was carried out using 4-[^18^F]fluorobenzaldehyde. This approach using the chemoselective oxime formation for labeling clearly reduced the synthesis time of this radiotracer compared to [^18^F]Galacto-RGD and made the clinical routine production more feasible. In contrast to Galacto-RGD, which belongs to the family of tracer based on the cyclic pentapeptide c(RGDfV), Fluciclatide shows higher binding affinity for integrin *α*_*v*_ *β*_5_ than for integrin *α*_*v*_ *β*_3_ [18].

**Figure 2:**
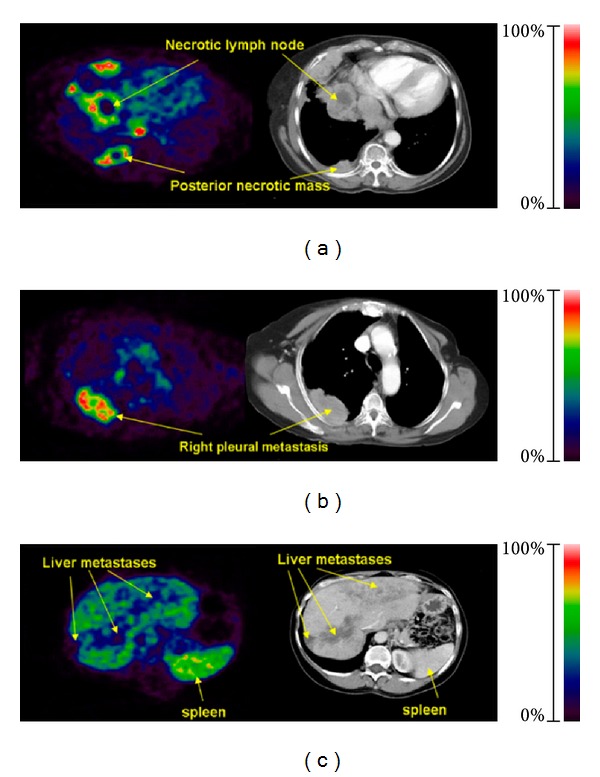
[^18^F]Fluciclatide PET: (a) patient with lung and pleural metastases. (b) Intralesion heterogeneity of uptake within pleural metastasis in PET image, which was not demonstrated as necrosis on corresponding CT section. (c) Liver metastases imaged as hypointense lesions because of high background signal (high uptake in spleen is possibly due to blood pooling) (with permission from Kenny et al. [18]).

In a study including 7 breast cancer patients it could be shown that all lesions found by CT could also be detected by [^18^F]Fluciclatide PET (Figure 2). In analogy to [^18^F]Galacto-RGD, a great variance in tracer uptake in the lesions was found with SUVs ranging from 2.0 to 40.0 [18]. Interestingly, metastases in the liver have been identified as regions of deficit uptake, because of the high background activity in normal liver tissue. Stability studies in vivo showed 74% intact tracer after 60 min in blood. Biodistribution and dosimetry studies in 8 healthy volunteers showed predominately renal excretion with the highest uptake in liver, combined walls of the intestine, and kidneys [58]. The compound was well tolerated with no drug-related adverse events reported. The mean effective dose was 0.026 mSv/MBq comparable to [^18^F]Galacto-RGD. An advantage of [^18^F]Fluciclatide compared with [^18^F]Galacto-RGD is the easier availability. However, further clinical studies are needed to demonstrate the potential of this compound for imaging integrin *α*_*v*_ *β*_3_/*α*_*v*_ *β*_5_ expression. Anyway, preclinical studies in mice already showed that monitoring of tumor response to an antiangiogenic sunitinib therapy using [^18^F]Fluciclatide-PET is feasible [59].

#### 2.1.3. [^18^F]RGD-K5

RGD-K5 is a closely related derivative to Galacto-RGD. The used cyclic pentapeptide c(RGDfK) and the sugar amino acid are identical for both compounds. The difference is found in the conjugation of 2-azidoacetic acid to the amino function of the sugar amino acid of RGD-K5 allowing labeling via “click chemistry” using 5-[^18^F]fluoro-1-pentyne. Similar to the labeling strategy using oxime formation for labeling, the click chemistry approach also reduced the overall synthesis time compared with [^18^F]Galacto-RGD, thereby increasing the availability of [^18^F]RGD-K5 [30].

Initial preclinical studies showed high affinity for integrin *α*_*v*_ *β*_3_ and predominantly renal elimination and high plasma stability in mice [60, 61]. This was confirmed by biodistribution and radiation dosimetry studies in monkeys and four healthy volunteers [62]. Organs with the highest activity concentration were bladder, kidneys, gallbladder, and liver. It was found that the plasma clearance half-life was approximately 12 min and that approximately 44% of the injected activity had been excreted in the urine by end of the study (~2.5 h). No clinical significant effects on vital signs had been found during the follow-up until 24 h after tracer injection. Depending on the bladder-voiding model the mean effective dose calculated was between 0.015 and 0.031 mSv/MBq and thus in the range of the other RGD tracers already in clinical studies. In an initial study with 12 breast cancer patients, [^18^F]RGD-K5 PET was compared with [^18^F]FDG-PET [63]. Out of 157 lesions detected using [^18^F]FDG, 122 lesions could be visualized by [^18^F]RGD-K5. In most lesions, [^18^F]FDG uptake was higher as found for [^18^F]RGD-K5 with no correlation between the uptake of the two compounds, confirming the results already found with other RGD tracers.

#### 2.1.4. [^68^Ga]NOTA-RGD

[^68^Ga]NOTA-RGD is the first ^68^Ga-labeled *α*_*v*_ *β*_3_ integrin-targeting compound for which initial clinical data are available. Due to the increasing availability of corresponding ^68^Ge/^68^Ga generators, this PET isotope becomes an interesting alternative to ^18^F especially for radiolabeling of peptides (see also below). NOTA-RGD is produced by conjugating SCN-Bz-NOTA to the amino function of the lysine in the cyclic pentapeptide c(RGDyK) [34]. The chelator forms very stable complexes with ^68^Ga, allowing labeling in short reaction times even at room temperature. The compound showed high affinity for the integrin *α*_*v*_ *β*_3_ in in vivo binding assays and rapid predominantly renal excretion with good tumor-to-background ratios in murine tumor models [34].

A biodistribution and radiation dosimetry study with 10 patients with lung cancer or lymphoma confirmed the excretion route with the highest activity found in kidneys and urinary bladder [64]. Comparably high radioactivity was also found in the liver. The effective dose was between 0.021 and 0.025 mSv/MBq depending on the calculation model and the voiding interval. Although tumor patients were included in this study, no information concerning tumor uptake was found. Anyway, in a preliminary study with six patients with liver metastases of a colorectal carcinoma in three out of the six patients increased [^68^Ga]NOTA-RGD uptake in the liver lesions could be detected [65]. Moreover, the patients who showed [^68^Ga]NOTA-RGD uptake revealed partial response after an antiangiogenic therapy with FOLFOX and bevacizumab, whereas the other half showed stable or progressive disease.

#### 2.1.5. [^18^F]Alfatide

**Figure 3:**
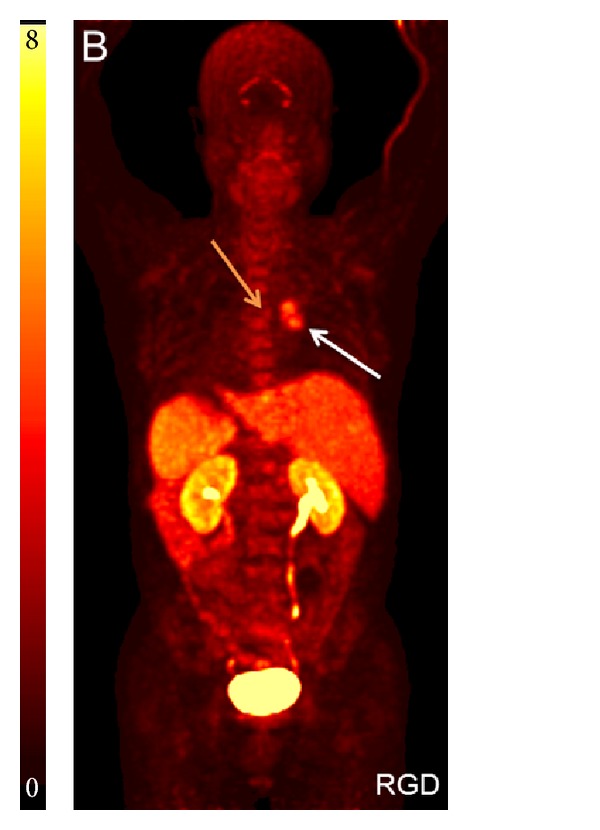
[^18^F]Alfatide PET: maximum intensity projection imaging of a patient with primary squamous carcinoma (white arrow) and lymph node metastasis (yellow arrow) (with permission from Wan et al. [19]).

Attempts optimizing the strategies in labeling peptides with ^18^F led to the introduction of ^18^F-aluminum fluoride [66]. This compound behaves similarly to radiometals concerning formation of complexes with, for example, NOTA derivatives introducing the advantage of using much faster and easier labeling protocols than those needed for ^18^F-labeling using prosthetic group strategies. The first compound of this class of tracer studied in patients is the ^18^F-labeled dimeric RGD-peptide [^18^F]AlF-NOTA-PRGD2 ([^18^F]Alfatide) [67]. It includes, besides the two cyclic RGD peptides c(RGDyK) bridged via a lysine, a PEG moiety as pharmacokinetic modifier and a Bz-NOTA moiety for complexation of “[^18^F]AlF.” In a pilot study including nine patients with lung cancer, [^18^F]Alfatide allowed identification of all tumors with SUVs of 2.9 ± 0.1 indicating a lower variance in tumor uptake as found by most other studies using RGD-derivatives in patients [19]. Major uptake was found in kidneys and bladder indicating renal excretion. Liver, spleen, and intestine showed comparable uptake as found in the tumor (Figure 3). Kinetic modeling based on dynamic PET scans suggested specific binding of the tracer. Moreover, immunohistochemical staining confirmed *α*_*v*_ *β*_3_ expression on both the tumor cells and the neovasculature of the squamous carcinoma patients.

### 2.2. Recent Tracer Developments for Imaging Integrin *α*_*v*_ *β*_3_/*α*_*v*_ *β*_5_ Expression

Since the first radiotracer for imaging integrin *α*_*v*_ *β*_3_ has been introduced in 1999 [46], a great variety of different derivatives have been described and a selection of optimization strategies have been introduced including optimization of the pharmacokinetics (e.g., glycosylation and PEGylation), the binding affinity (multimerization), and the labeling strategies. There are already a range of reviews dealing with the different aspects (e.g., [6, 8]). Here, we focus on the most recent approaches in introducing new or optimized labeling strategies.

#### 2.2.1. ^68^Ga-Labeled Derivatives

Preclinical as well as clinical data demonstrated successful noninvasive determination of integrin *α*_*v*_ *β*_3_ expression with [^18^F]Galacto-RGD PET (see [17, 49, 50, 52] and above). The major drawback of this compound is the complex and time consuming labeling strategy using [^18^F]fluoropropionic acid as prosthetic group. One strategy to overcome this problem is based on the introduction of ^68^Ga. Due to the increasing amount of commercially available ^68^Ga/^68^Ge generators [68], this isotope becomes an interesting alternative to ^18^F, especially when peptide labeling is considered. Direct labeling of peptides modified with the corresponding chelator systems with ^68^Ga avoids the time consuming preparation of prosthetic groups usually needed for labeling peptides with ^18^F.

First approaches to introduce ^68^Ga-labeled RGD peptides are focused on the use of DOTA-conjugated RGD peptides. [^68^Ga]DOTA-RGD showed high affinity for the integrin *α*_*v*_ *β*_3_ in in vitro binding studies and receptor selective tracer accumulation in a murine tumor model [35]. However, high protein bound activity was also found compared to the ^111^In-labeled analog. The high plasma protein binding leads to increased activity concentration in blood and to inferior imaging properties compared with [^18^F]Galacto-RGD. Although DOTA is successfully used in DOTA-TOC and derivatives for binding of ^68^Ga, it is known that the cyclododecane ring of DOTA does not have the optimal size for complexing gallium [69]. A more favorable chelating system is the NOTA system, which contains a nine-membered ring more suitable for binding ^68^Ga. This system was initially introduced with NOTA-RGD [34] and NODAGA-RGD [36, 70]. The later showed significantly reduced binding to plasma proteins compared to [^68^Ga]DOTA-RGD resulting in equal imaging properties in a murine tumor model as found for [^18^F]Galacto-RGD. Moreover, due to the high complex binding constant labeling of NODAGA-RGD can be carried out at room temperature with low amounts of peptide in high radiochemical yield and purity. Based on these positive results initial clinical studies are most recently started.

The last few years, alternative chelating systems have been introduced for ^68^Ga-labeling of RGD peptides. This include RGD peptides conjugated to H_2_dedpa derivatives [38] and TRAP(RGD)_3_ [20]. Based on the H_2_dedpa scaffold a monomeric and a dimeric tracer have been introduced (H_2_-RGD-1 and H_2_-RGD-2). Both compounds showed rapid ^68^Ga-labeling at room temperature in high radiochemical yield. The complexes were stable if challenged with transferrin and showed IC_50_ values determined using a competitive cell binding assay of approximately 2.4 *μ*M for the monomeric H_2_-RGD-1 and approximately 0.2 *μ*M for the dimeric H_2_-RGD-2. Anyway, in biodistribution as well as small animal PET studies high activity concentration was found in blood even 2 hours after injection making these compounds uncompetitive with the already introduced ^68^Ga-labeled derivatives. Although no log *P* values are described, it is assumed that the aromatic components of the chelating systems increase the lipophilicity which might be the reason for this finding.

**Figure 4:**
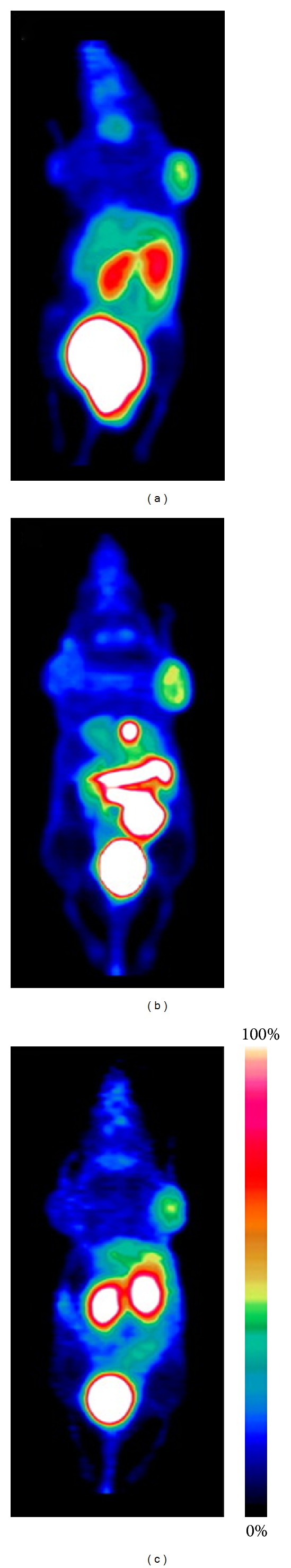
[^68^Ga]TRAP(RGD)_3_: comparison of maximum intensity projections of microPET scans of the same M21/M21L human melanoma xenografted mouse (a) [^68^Ga]TRAP(RGD)_3_, (b) [^18^F]Galacto-RGD, (c) [^68^Ga]NODAGA-RGD (scaling adapted to show equal intensities in M21 tumors and background. Scale indicates percentage of the maximum displayed signal level) (with permission from Notni et al. [20]).

The TRAP chelator uses the similar nine-membered ring system as found in NOTA but possesses phosphinic acid groups instead of the carboxylic acid groups. This modification results in two advantages: (a) due to the high binding affinity of the chelator for gallium it allows labeling with very low amounts of TRAP-modified peptides and (b) due to the additional functionality of the phosphinic acid it allows direct conjugation of up to three targeting peptides per chelating system, making it an advantage system for introducing the multimerization approach. Based on these results, the trimeric TRAP(RGD)_3_ was introduced [20]. This compound demonstrated rapid labeling using low peptide amounts, resulting in specific activities of up to 1 TBq/*μ*mol, very high binding affinity for the integrin *α*_*v*_ *β*_3_ in a competitive cell binding assay, and good tumor/background ratios in a murine tumor model. Anyway, direct comparison of the biodistribution data in the murine M21/M21-L tumor model with [^68^Ga]NODAGA-RGD 90 min after injection showed comparable values for both compounds indicating that, despite better performance in vitro, the in vivo effect is negligible (Figure 4). Most recently, [^68^Ga]NOPO-RGD was introduced [37]. This chelator belongs to the “TRAP family” with the known advantages of fast complexation kinetics, high stability, and extremely high resulting specific activity. Major difference is found in the fact that only one phosphinic acid group is functionalized for conjugation to peptides. Thus, multimeric compounds cannot be produced. But the additional hydroxymethyl groups increase the polarity of any conjugated peptide and may improve renal elimination.

#### 2.2.2. RGD Peptides Labeled with ^18^F via Click Chemistry Approaches

After the Cu(I)-catalyzed azide-alkyne 1,3-cycloaddition (CuAAC) reaction (better known as the most prominent example of “click chemistry”) was introduced for radiolabeling with ^99*m*^Tc in 2006 [71], this technique was also applied for ^18^F-labeling of RGD peptides. The apparent advantages of the CuAAC reaction are mainly reflected by their high yield under mild conditions, its chemoselectivity, and the formation of 1,2,3-triazole with similar polarity and size as found in an amide bond [72]. Most importantly, for peptide labeling, there are no interferences with common functionalities found in amino acid side chains. These aspects make click chemistry based approaches an interesting alternative to common prosthetic group techniques for labeling peptides with ^18^F, as highlighted by the reviews of Kettenbach et al. [73] and Maschauer and Prante [74] within this special issue. In general, there are two possible approaches for the CuAAC reaction: either a ^18^F-labeled organoazide or a ^18^F-labeled alkyne is used as prosthetic group.

In a preliminary study, a dimeric RGD peptide was modified with an azide and as prosthetic group a ^18^F-fluoro-PEG-alkyne derivative was used [31]. The product could be achieved in good radiochemical yield. Anyway, this procedure includes two HPLC separation steps, rendering it unfavorable compared to other prosthetic group labeling techniques. Glaser et al. compared the ^18^F-labeling of RGD peptides via oxime formation, click labeling, and S-alkylation [29]. The prosthetic groups include [^18^F]fluorobenzaldehyde, 2-[^18^F]fluoroethylazide, and [^18^F]fluoropropanethiol. It was concluded that the click labeling resulted in comparable yields as found for the fluorobenzaldehyde approach without the need for purification of the prosthetic group. However, 2-[^18^F]fluoroethylazide seems to be too small to be separated from the labeled RGD peptide. For the synthesis of [^18^F]RGD-K5, [^18^F]fluoropentyne was used as prosthetic group. With an optimized protocol for radiosynthesis the peptide could be labeled within 70 min with 35% radiochemical yield (EOB) [30]. Due to the good preclinical performance, this compound is already studied in patients (see also above).

Introduction of sugar derivatives as pharmacokinetic modifier has successfully been introduced with [^18^F]Galacto-RGD [47] and was later also used with [^18^F]RGD-K5 [30]. Maschauer et al. combined the click labeling approach with the introduction of sugar derivatives allowing labeling as well as pharmacokinetic optimization in one step [32, 75, 76]. Four different sugar azides have been used as prosthetic groups, including glucose, galactose, maltose, and cellobiose derivatives, which were conjugated via propargylglycine to the modified RGD peptide. The overall synthesis time was in the range of 70–75 min with decay-uncorrected radiochemical yields between 16% and 24%. A favorable performance was found for [^18^F]Mlt-RGD, revealing comparable tumor-to-background ratios as found for [^18^F]Galacto-RGD with the advantage of a more rapid and simplified radiosynthesis [32].

#### 2.2.3. ^18^F/^19^F Isotopic Exchange and ^18^F-Fluoride Aluminum Complexes for Labeling RGD Peptides

Despite a great variety of studies focused on the optimization of ^18^F-labeling of RGD peptides including some approaches with improved labeling conditions compared to [^18^F]Galacto-RGD, none of the newly introduced prosthetic group approaches can compete with the simple and rapid labeling strategies based on ^68^Ga. Thus, alternative ^18^F-labeling approaches have been studied for labeling RGD peptides including isotopic exchange strategies using silicon fluoride acceptors (SiFA) [33] or arylfluoroborates [21] as well as complexation of an ^18^F-aluminum fluoride species (AlF) [77].

**Figure 5:**
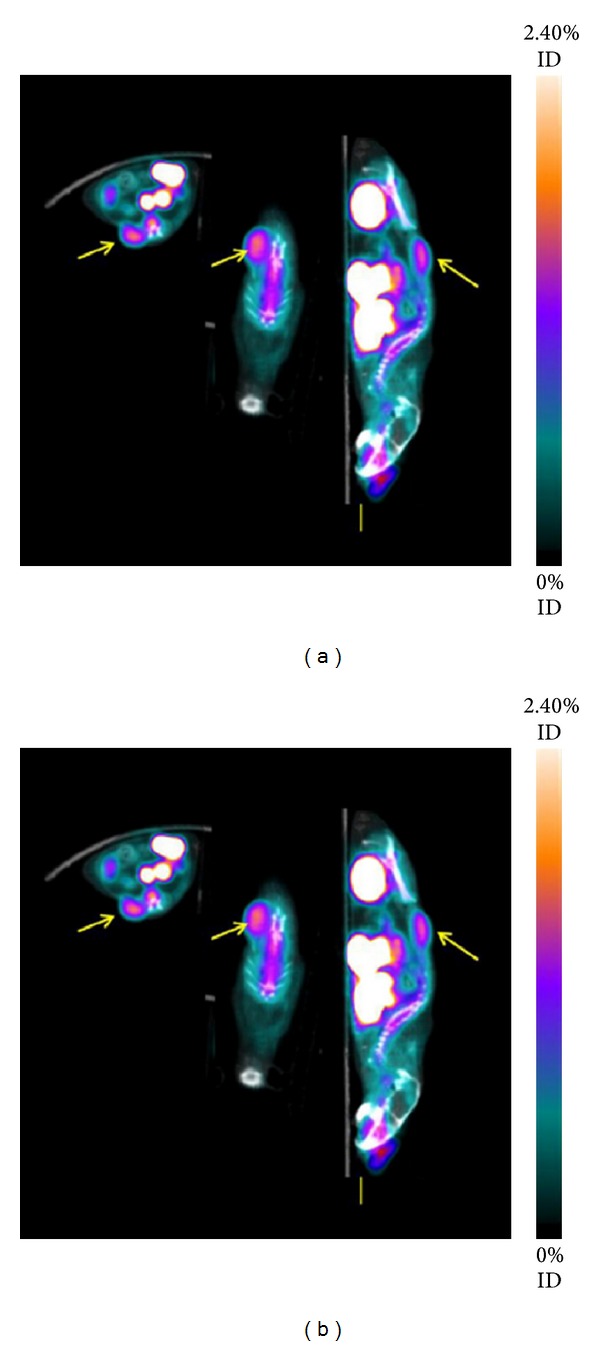
RGD-[^18^F]ArBF_3_^−^: PET/CT images of (a) an unblocked and (b) a blocked mouse. Arrow marks the tumor in three perspectives (with permission from Liu et al. [21]).

The SiFA method is based on ^18^F-labeling of p-(di-tert-butylfluorosilyl) benzaldehyde. It has been shown that this labeling precursor allows isotopic exchange in almost quantitative yields, resulting in unexpected high specific activities [33], which are even higher as specific activities found for peptides labeled via conventional n.c.a. ^18^F-labeling techniques, without HPLC purification. Conjugation of the prosthetic group was carried out via oxime formation using an aminooxy modified cyclic RGD peptide. Altogether, this results in cyclo (fK([^18^F]SiFA-AO-N)RGD) in high radiochemical yield within approximately one hour. In vitro and in vivo evaluation of the compound still remains to be elucidated to demonstrate the imaging properties of this RGD derivative. However, a highly lipophilic precursor is needed for this labeling technique, which might negatively influence the pharmacokinetics of the radiolabeled peptides. Another strategy using radiolabeling by isotopic exchange is based on boron derivatives. It was shown that kit-like ^18^F-labeling resulting in an [^18^F]aryl trifluoroborate-containing RGD peptide is feasible in high specific activity in reaction times below one hour [21]. Initial small animal PET data showed high activity concentration in bladder indicating predominantly renal elimination (Figure 5). However, despite high specific activity tracer accumulation in a murine U87MG glioblastoma model was comparably low; thus, further studies are needed to finally access the quality of this kind of tracer for imaging integrin *α*_*v*_ *β*_3_ expression.

Recently, a technique to produce the ^18^F-aluminum fluoride species (Al^18^F)^2+^ has been introduced [66] and has shown that this compound forms stable complexes with the NOTA ligand conjugated to peptides. After optimization [78], this technique allows labeling of peptides in a one-step synthesis without HPLC purification in analogy to radiometal labeling with, for example, ^68^Ga or ^64^Cu. Based on these developments, [^18^F]Alf-NOTA-RGD_2_ has been introduced [77]. In this case, labeling including HPLC could be carried out in 40 min. In a cell binding study, the compound showed comparable IC_50_ values as found for the dimeric lead structure and high tumor uptake and rapid elimination from the body in a murine tumor model. Comparison of [^18^F]AlF-NOTA-PRGD_2_, which differs in an additional PEG linker from the initial compound, with a dimer labeled with ^18^F via fluoropropionic acid as prosthetic group and a dimer labeled with ^68^Ga using small animal PET showed comparable pharmacokinetics and quantitative parameters for all three compounds [79]. Based on this data, the so-called [^18^F]Alfatide is already studied in initial clinical trials (see also above). Subsequently, the influence on different linker was studied and the labeling protocol was optimized [80]. The replacement of the HPLC separation by C-18 cartridge purification allowed production of the compound with good radiochemical yield and high radiochemical purity within 30 min. The compounds were stable in mouse serum up to 120 min and the highest binding affinity using a cell binding assay as well was found for NOTA-E[PEG_4_-c(RGDfK)]_2_. However, in vivo studies using a murine glioblastoma model could not confirm the in vitro findings. The biodistribution data demonstrated comparable tumor uptake for NOTA-E[c(RGDfK)]_2_ and NOTA-E[PEG_4_-c(RGDfK)]_2_ but slightly better tumor-to-background ratios are found for the latter.

## 3. Tracer Targeting Integrins *α*_5_ *β*_1_ and *α*_*v*_ *β*_6_

As already mentioned, most work on the development of tracer for imaging integrins is dedicated to the development of compounds targeting the integrins *α*_*v*_ *β*_3_ and *α*_*v*_ *β*_5_. Recently, additional integrins came into the focus of interest. These include the integrins *α*_5_ *β*_1_ and *α*_*v*_ *β*_6_.

### 3.1. Integrin *α*_5_ *β*_1_

**Figure 6:**
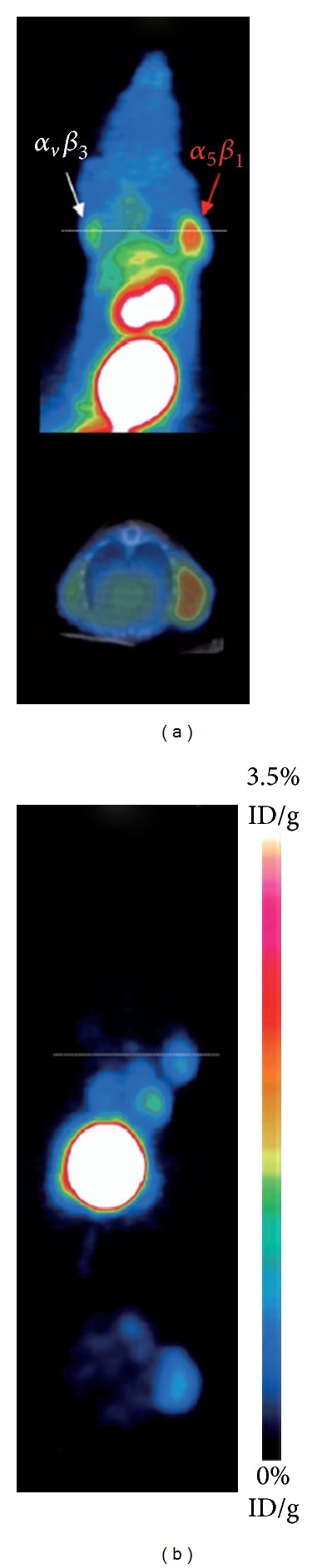
[^68^Ga]*α*_5_ *β*_1_-ANT: maximum intensity projection images (MIP) of microPET scans. Upper row: mice bearing RKO (*α*_5_ *β*_1_-positive) and M21 (*α*_*v*_ *β*_3_-positive)tumor xenografts on right and left shoulder, respectively, (white arrow: M21; red arrow: RKO). Lower row: axial slices corresponding to the white line in upper row MIP images. (a) Injection of [^68^Ga]*α*_5_ *β*_1_-ANT. (b) Blocking experiment (with permission from Neubauer et al. [22]).

Heckmann et al. [81] developed based on tyrosine and azaglycine scaffolds nonpeptide antagonists of the integrin *α*_5_ *β*_1_. Comprehensive structure activity relationship studies including docking experiments with a homology model resulted in azaglycine derivatives with low nanomolar affinity for *α*_5_ *β*_1_ and up to 10^4^-fold higher selectivity when compared with *α*_*v*_ *β*_3_. The superior properties of the azaglycine derivatives compared with the tyrosine scaffold based compounds may result from enhanced rigidity of the first. Based on this data, one of the most promising azaglycine derivatives was modified by conjugation of NODAGA to the alkoxy benzoic acid moiety of the *α*_5_ *β*_1_ antagonist [22]. A competitive solid phase integrin binding assay demonstrated that this modification had no influence on binding affinity and selectivity to integrin *α*_5_ *β*_1_. A murine tumor model of mice bearing an *α*_5_ *β*_1_-positive human colon carcinoma (RKO) on the one flank and an *α*_*v*_ *β*_3_-positive human melanoma (M21) on the other flank confirmed receptor specific uptake and allows visualization of the *α*_5_ *β*_1_-positive tumor only (Figure 6).

A common approach to search for biological active peptides is based on phage display libraries. Screening a CX7C library including a random heptapeptide sequence flanked by two cysteine for high affinity integrin *α*_5_ *β*_1_ binder resulted in the peptide H-Cys*-Arg-Arg-Glu-Thr-Ala-Trp-Ala-Cys*-OH (H-C*RRETAWAC*-OH) [82]. This peptide was used as lead structure for the development of a ^18^F-labeled derivative for noninvasive imaging of integrin *α*_5_ *β*_1_ expression (more detailed information will be found in this special issue under Haubner et al. “*H-CRRETAWAC-OH, a lead structure for the development of radiotracer targeting integrin α*_5_ *β*_1_ *?*” [39]). Briefly, for labeling, 2-[^18^F]fluoropropionic acid was used as prosthetic group. With an isolated receptor binding assay it was demonstrated that modification of the lead structure reduced binding to integrin *α*_5_ *β*_1_ by a factor of 10. Comparison of the binding affinity for *α*_5_ *β*_1_, *α*_*v*_ *β*_3_, and *α*_IIb_ *β*_3_ revealed that selectivity was not affected. Despite high affinity for the integrin and stability in human serum in vivo, biodistribution data of [^18^F]FProp-C*RRETAWAC*-OH using a murine tumor model were disappointing. In fact, the highest tracer accumulation was found for the tumor, but similar high radioactivity concentration was found in blood. Additionally, activity concentration in the organs remains almost constant over the observation period of 120 min leading to tumor-to-background ratios between 1 and 2, making this compound not suitable for imaging integrin *α*_5_ *β*_1_ expression.

### 3.2. Integrin *α*_*v*_ *β*_6_

The most prominent lead structure for the development of radiotracer for imaging integrin *α*_*v*_ *β*_6_ is the 20-amino acid peptide A20FMDV2 (sequence: NAVPNL**RGDLQVL**AQKVART). The sequence is derived from the GH loop of an envelope protein of the foot-and-mouth diseases virus (FMDV) [83] which mediates FMDV infection via binding to the integrin *α*_*v*_ *β*_6_ [84, 85]. The central binding region includes the RGD sequence followed by an LXXL motif, where X specifies variable amino acids. Phage display libraries indicate that the DLXXL sequence is responsible for the high *α*_*v*_ *β*_6_ specificity [86].

**Figure 7:**
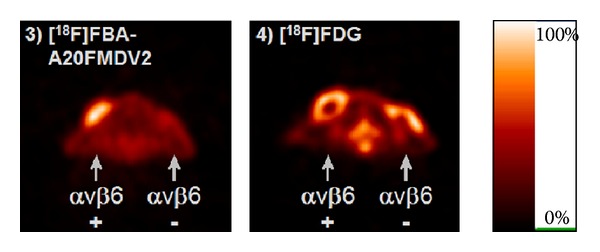
[^18^F]FBA-A20FMDV2: (3) representative transaxial microPET 45–60 min after injection. The positive (*α*_*v*_ *β*_6_-expressing DX3puro*β*6) tumors were located near the left shoulder and the negative (control DX3puro) tumors near the right shoulder. For comparison, (4) depicts a [^18^F]FDG scan of the animal shown in (3), obtained within 5 d. (with permission form Hausner et al. [23]).

This peptide was initially labeled with a [^18^F]fluorobenzoyl group via a solid-phase labeling strategy [23]. In a competitive binding ELISA including integrin *α*_*v*_ *β*_6_, *α*_*v*_ *β*_3_, *α*_*v*_ *β*_5_, and *α*_5_ *β*_1_ it was demonstrated that the N-terminal modification has no influence on binding affinity and selectivity. Evaluation of the tracer using a murine tumor model including *α*_*v*_ *β*_6_-positive (DX3puro) and *α*_*v*_ *β*_6_-negative (DX3puro*β*6) xenografts demonstrated receptor selective uptake of [^18^F]FBA-A20FMDV2 (Figure 7) [23]. However, uptake and retention in the tumor were comparably low, which might be due to the low metabolic stability of the compound. To improve the stability and the pharmacokinetic behavior, polyethylene glycol (PEG) moieties have been introduced. This resulted in [^18^F]FBA-PEG_28_-A20FMDV2 and [^18^F]FBA-(PEG_28_)_2_-A20FMDV2 [40]. HPLC analysis of mouse urine samples showed increased stability of the PEGylated compounds with only one major metabolite detected. Also tumor retention could be significantly improved with almost constant uptake up to 4 h after injection. However, also retention in other organs has been increased. In particular, the introduction of a second PEG_28_ unit was not beneficial due to the resulting high uptake and retention in the kidneys. Most recently, Hausner et al. [41] evaluated the copper-free, strain-promoted click chemistry for ^18^F-labeling of A20FMDV2. This modified click chemistry approach should eliminate the need for potentially toxic copper catalysts. The radiotracer was readily prepared with high radiochemical purity, but the required cyclooctyne derivative introduces a very lipophilic moiety which negatively influences the pharmacokinetic of the resulting [^18^F]FBA-C_6_-ADIBON_3_-PEG_7_-A20FMDV2. Thus, despite receptor specific binding and good metabolic stability, the tumor uptake was low and the radioactivity concentration in urine as well as gall bladder was very high, indicating both renal and hepatobiliary elimination making this compound not suitable for imaging integrin *α*_*v*_ *β*_6_ expression.

Additional approaches are based on the introduction of chelating systems for labeling with ^111^In-indium or ^64^Cu-copper. For ^111^In-labeling, DTPA was conjugated to the N-terminal end of the peptide [42]. DTPA conjugation has no effect on peptide binding affinity and receptor specificity. Serum stability was comparable as found for [^18^F]FBA-A20FMDV2 with several metabolites found after 4 h incubation. Despite comparable low stability, tumor uptake was higher as found for the ^18^F-labeled derivative. If this could be ascribed to the different tumor models used or to a better performance of the [^111^In]DTPA-A20FMDV2, it has to be figured out by direct comparison in the same animal model. Extremely high radioactivity concentration was found in kidneys at 1 hour after injection. Other organs with comparable uptake as found in the tumor are lower gastrointestinal tract, gall bladder, and stomach. This seems to be due to expression of the integrin *α*_*v*_ *β*_6_ in these organs, which were examined by immunohistochemical staining of the corresponding paraffin-embedded murine tissue and confirmed by blocking studies. High-resolution SPECT of mice demonstrate clear visualization of *α*_*v*_ *β*_6_-expressing tumors but also indicate high activity concentration in kidneys and bladder. [^111^In]DTPA-A20FMDV2 was also used to study imaging of *α*_*v*_ *β*_6_ integrin for molecular stratification of idiopathic pulmonary fibrosis [87]. It could be demonstrated that levels of [^111^In]DTPA-A20FMDV2 in the lung correlated positively with hydroxyproline, *α*_*v*_ *β*_6_ protein, and itgb6 messenger RNA levels indicating that this technique might be feasible to be used for stratifying therapy for patients with pulmonary fibrosis.

A study by Hu et al. [43] was designed to determine the best candidate out of four chelating systems to label PEG_28_-A20FMDV2 with ^64^Cu. This include a triazacyclononane derivative (NOTA), a tetraazacyclododecane derivative (DOTA), a tetraazabicyclo[6.6.2] hexadecane derivative (CB-TE1A1P), and a hexaazabicyclo[6.6.6]icosane derivative (BaBaSar). Independent of the chelating system, all compounds could be labeled under mild conditions in good radiochemical purity and specific activity. None of the chelating systems influenced the selectivity for the integrin *α*_*v*_ *β*_6_ in a cell binding assay. The lowest binding and internalization were found for [^64^Cu]NOTA-PEG_28_-A20FMDV2. Stability studies in mouse serum after 24 hours incubation revealed the highest amount of intact tracer for [^64^Cu]CB-TE1A1P-PEG_28_-A20FMDV2 (<45%) and the lowest for [^64^Cu]BaBaSar-PEG_28_-A20FMDV2 (14%). Initial biodistribution data did not present the best candidate. Although high positive-to-negative tumor uptake ratios were found for [^64^Cu]CB-TE1A1P-PEG_28_-A20FMDV2 and for [^64^Cu]BaBaSar-PEG_28_-A20FMDV2, there was significant higher kidney uptake as found for the other two tracers. Another unpredicted finding was that blocking resulted only for three compounds in a reduced uptake in the receptor-positive tumor. For [^64^Cu]NOTA-PEG_28_-A20FMDV2, an unexplained increase of tumor uptake was found. Altogether, this study demonstrated that ^64^Cu-labeling of A20FMDV2 derivatives is possible but much more detailed experiments that may be also including alternative chelating systems are necessary before a final decision about the best performer can be made.

**Figure 8:**
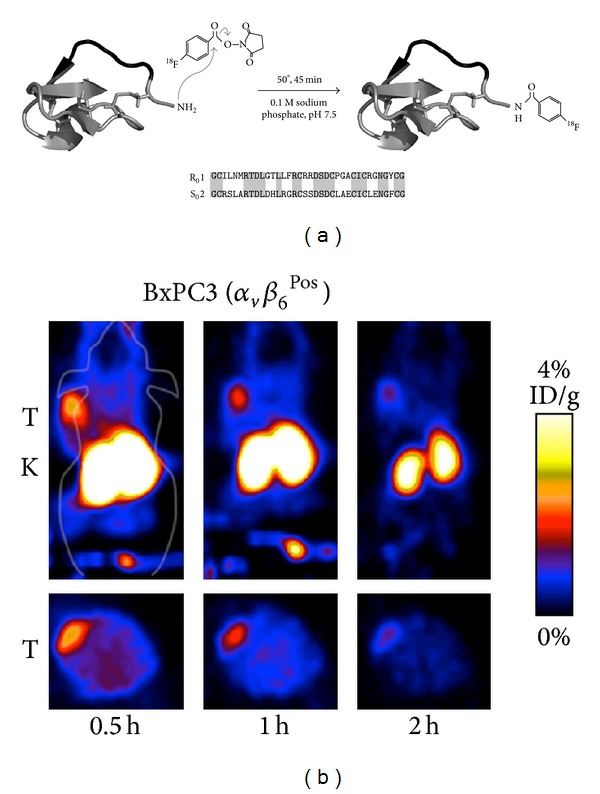
Cystine knot based tracer: (a) R_0_1 and S_0_2 are cystine knot peptides that contain 3 disulfide bonds, an active binding loop (black), and a sole primary amine at N terminus used for labeling via ^18^F-SFB. Peptide sequences are presented with conserved residues highlighted. (b) ^18^F-fluorobenzoate-R_0_1 small-animal PET imaging of BxPC3 pancreatic adenocarcinoma (integrin *α*_*v*_ *β*_6_-positive) bearing nude mice (five-minute static scans were acquired at 0.5, 1, and 2 h p.i.; decay-corrected coronal and transverse slices are presented; tumor (T) and kidneys (K) are marked on images) (with permission from Hackel et al. [24]).

To develop a more stable and effective agent for imaging integrin *α*_*v*_ *β*_6_ cysteine knot peptides were engineered which demonstrated nanomolar affinity for this integrin [44]. Four DOTA-derivatized compounds were labeled with ^64^Cu and metabolic stability was studied in mouse serum. Two derivatives ([^64^Cu]DOTA-S_0_2 and [^64^Cu]DOTA-E_0_2) showed high stability with more than 95% intact tracer after 24 hours incubation. In vivo biodistribution as well as small animal PET demonstrated receptor specific tumor uptake for all compounds tested but also extremely high activity concentration in kidneys for [^64^Cu]DOTA-R_0_2, [^64^Cu]DOTA-E_0_2, and [^64^Cu]DOTA-R_0_1. In a further study R_0_1 and S_0_2, were labeled via N-succinimidyl-4-^18^F-fluorobenzoate (Figure 8) [24]. In particular, ^18^F-fluorobenzoate-S_0_2 showed high serum stability. Despite lower stability in the in vitro assay, tumor uptake was superior for ^18^F-fluorobenzoate-R_0_1. For both compounds, a clear reduction in kidney uptake was found when compared with the ^64^Cu-labeled analogs. Anyway, with 16% ID/g at 1 h p.i. it remains high especially for ^18^F-fluorobenzoate-R_0_1. However, the results from coinjection studies remained inexplicable. For ^18^F-fluorobenzoate-R_0_1, at least a slight reduction in tumor uptake was found, but no reduction was observed for ^18^F-fluorobenzoate-S_0_2. Most recently, S_0_2 was modified with a single amino acid chelator (SAAC) and labeled with ^99*m*^Tc (CO)_3_ [45]. Similar to the other cysteine knot derivatives, [^99*m*^Tc]SAAC-S_0_2 showed high metabolic stability and integrin *α*_*v*_ *β*_6_ specific uptake but biodistribution studies revealed, with exception of tumor-to-muscle ratio, that most of the tumor-to-organ ratios are approximately one or even clearly below one. Very high activity concentration is again found for the kidneys, independent of the use of the serine-rich derivative, which should avoid high kidney uptake.

## 4. Summary and Conclusion

Approximately 15 years ago, the first radiolabeled RGD peptides were introduced to image integrin *α*_*v*_ *β*_3_. Starting from the initially iodinated derivatives, a great variety of different compounds labeled with almost the whole set of available isotopes used in nuclear medicine tracer techniques have been described, but only a small set yet entered clinical trials. The first and most intensively studied one is [^18^F]Galacto-RGD which showed receptor selective tracer accumulation in the tumor with rapid predominantly renal elimination resulting in good tumor-to-background ratios and low radiation burden for the patient. The drawback of this compound is the complex time-consuming radiosynthesis. Thus, one major goal of the subsequently developed compounds was to optimize the radiolabeling strategy. One approach was focused on alternative ^18^F-labeling strategies including oxime formation, click chemistry, isotopic exchange labeling, and introduction of aluminum fluoride species. Another approach to develop new PET tracer was focused on the introduction of ^68^Ga as an alternative to ^18^F for PET imaging. Based on each of the described labeling strategies, at least one candidate RGD peptide has entered clinical studies, with the exception of the isotopic exchange labeling strategy. All approaches produce the radiopharmaceutical in shorter production times as described for [^18^F]Galacto-RGD, with most significant reductions found for the ^68^Ga-labeling approach followed by the aluminum fluoride approach. All tracers have in common that they allow receptor specific imaging of integrin *α*_*v*_ *β*_3_ (and *α*_*v*_ *β*_5_) expression, show rapid predominately renal excretion with low radiation burden, and are well tolerated. For most radiopharmaceuticals, a great variance in tracer uptake in the lesions is found. One exception is the dimeric tracer [^18^F]Alfatide. The initial study with nine patients resulted in very low variance in the SUV. However, a clinical study directly comparing different RGD tracers is lacking; thus, a final conclusion which compound performs best is not possible, yet. However, as there are already a variety of clinical studies using radiolabeled RGD peptides demonstrating their feasibility for imaging *α*_*v*_ *β*_3_, it is now of utmost importance to study how patients can benefit from this PET imaging approach. Therefore, further studies have to demonstrate whether corresponding antiangiogenic therapies can be controlled using this imaging technique. Most recently, alternative applications are also studied including assessment of plaque inflammation. However, again more comprehensive studies are needed allowing a final conclusion. In parallel to the radiopharmaceuticals already in clinical studies, a set of new compounds and strategies are evaluated. Among this set of candidates, several may enter clinical trials soon, including [^68^Ga]NODAGA-RGD and [^68^Ga]TRAP(RGD)_3_.

In addition to the integrins *α*_*v*_ *β*_3_/*α*_*v*_ *β*_5_ the integrins *α*_5_ *β*_1_ and *α*_*v*_ *β*_6_ recently came into the focus of interest. Integrin *α*_5_ *β*_1_ might even be more important in the angiogenic process as the integrin *α*_*v*_ *β*_3_; thus, initial tracer either based on nonpeptidic scaffolds or on results from screening phage display libraries has been developed. The performance of the latter was not sufficient to be used for imaging integrin *α*_5_ *β*_1_ whereas the nonpeptide derivatives seem to be promising and are the basis for further studies. Integrin *α*_*v*_ *β*_6_ does not seem to be involved in angiogenesis but was found to be highly expressed on a variety of tumors. Moreover, expression seems to correlate with pure outcome; thus, this integrin was also used as target structure for the development of radiopharmaceuticals. In the present days, two lead structures are studied. One is based on the sequence of a loop of an envelope protein of the foot-and-mouth diseases virus and the other is based on cystine knots. Both classes of compounds were radiolabeled with different isotopes, including ^18^F and ^64^Cu, and revealed receptor-specific binding in vitro and in vivo. However, on the one hand, some of the tracers lack metabolic stability and, on the other hand, tracer excretion is not optimal, leading to high activity in a variety of organs including the kidneys as the dose-limiting organ. Thus, although initial data demonstrate that *α*_*v*_ *β*_6_-specific imaging is feasible, further optimizations are needed to find suitable compounds for noninvasive imaging of this receptor in patients.

## Figures and Tables

**Table 1:** Summary of the most important RGD peptide tracers discussed.

Compound name	Used peptide sequence	“Labeling species”	Total prod. time	Labeling yield/spec act	Reference
**α**_**v**_ **β**_3_/**α**_**v**_ **β**_5_ **targeting**					

[^18^F]Galacto-RGD	**c(RGDfK)**	**nitrophenyl-2-**[^18^ **F**]**fluoropropionate** via amide formation	200 min	29 ± 5% (dc^7^) 40–100 GBq/µmol	[28]
	Lysine-NH_2_ SAA^1^ modified		

[^18^F]Fluciclatide	-^∗1^ **CH**_2_ **-CO-KC** ^∗2^ **RGDC** ^∗2^ **FC** ^∗1^-	**p-**[^18^ **F**]**fluorobenzaldehyde** via oxime formation	75 min	40 ± 12% (dc) 173 ± 52 GBq/µmol	[29]
	^∗1^Thioether bridge ^∗2^Disulfide bridge C-terminal PEG modified Lysine-NH_2_ PEGylated and aminooxy derivatized		

[^18^F]RGD-K5	**c(RGDfK)**	**5-[** ^ 18^ **F]fluoro-1-pentyne** via “click chemistry”	75 min	35% (dc) 100–200 GBq/µmol	[30]
	Lysine-NH_2_ SAA modified SAA N_3_-functionalyzed		

[^18^F]FPTA-RGD2	**Dimeric c(RGDyK)**	**[** ^ 18^ **F]fluoro-PEG3-alkyne** via “click chemistry”	110 min	54% (dc) 100–200 GBq/µmol	[31]
	Lysine-NH_2_ used for dimerization Bridged via glutamic acid Derivatized with 5-azidopentanoic acid		

[^18^F]Mlt-RGD	**c(RGDfPra)** ^ 4^	**6**′**-deoxy-6**′**-[** ^18^ **F]fluoro-*β*-maltosyl azide** via “click chemistry”	75 min	24% (ndc) 50–200 GBq/µmol	[32]
			

c(fK([^18^F]SiFA-AO-N) RGD)	**c(RGDfK)**	**p-(di-tert-butyl-[** ^ 18^ **F]fluorosilyl)-benzaldehyde** ^ 5^ via oxime formation	40 min	50–55% (ns^8^) 225–680 GBq/µmol	[33]
	Lysine-NH_2_ aminooxy acetic acid derivatized			

RGD-[^18^F]ArBF_3_^−^	**c(RGDfK)**	**[** ^ 18^ **F]fluoride** isotopic exchange	35 min	65% (dc) 518 GBq/µmol	[21]
	Lysine-NH_2_ 1-succinyl-4-(2-Trifluoroboryl-1,3,5-trifluorobenzoyl)-piperazine derivatized				

[^18^F]Alfatide	**Dimeric c(RGDyK)**	[^18^ **F**]**aluminum fluoride species** via complexation	20 min	42% (dc) 37 GBq/µmol	[19]
	Lysine-NH_2_ used for dimerisation Bridged via glutamic acid PEG linker and NOTA for complexation		

[^68^Ga]NOTA-RGD	**c(RGDyK)**	^ 68^ **Ga**	10 min^2^	89% (ns)	[34]
	Lysine-NH_2_ SCN-Bz-NOTA conjugated	via complexation		18 GBq/µmol	

[^68^Ga]DOTA-RGD	**c(RGDfK)**	^ 68^ **Ga**	7 min^2^	>95% (ns)	[35]
	Lysine-NH_2_ DOTA conjugated	via complexation		—	

[^68^Ga]NODAGA-RGD	**c(RGDfK)**	^ 68^ **Ga**	5 min^2^	>96% (ns)	[36]
	Lysine-NH_2_ NODAGA conjugated	via complexation		10–20 GBq/µmol	

[^68^Ga]TRAP(RGD)_3_	**Trimeric c(RGDfK)**	^ 68^ **Ga** via complexation	5 min^2^	— 0.8–1 TBq/µmol	[20]
	Lysine-NH_2_ TRAP conjugated Chelator PEG modified Monomers linked via chelator		

[^68^Ga]NOPO-RGD	**c(RGDfK)**	^ 68^ **Ga**	15 min	94% (dc)	[37]
	Lysine-NH_2_ NOPO conjugated	via complexation		1.4 TBq/µmol	

[^68^Ga-(RGD-1)]^+^	**c(RGDyK)**	^ 68^ **Ga**	10 min^2^	97% (ns)	[38]
	Lysine-NH_2_ H_2_dedpa conjugated	via complexation		34 GBq/µmol	

[^68^Ga-(RGD-2)]^+^	**Dimeric c(RGDyK)**	^ 68^ **Ga** via complexation	10 min^2^	99% (ns) 25 GBq/µmol	[38]
	Lysine-NH_2_ H_2_dedpa conjugated monomers linked via chelator		

**α**_5_ **β**_1_ **targeting**					

[^68^Ga]*α*_5_ *β*_1_-ANT	**Nonpeptide RGD mimetic**	^ 68^ **Ga**	—	—	[22]
	Conjugated via linker with NODAGA	via complexation			

[^18^F]FProp-CRRETAWAC-OH	H-*CRRETAWAC*-OH	**nitrophenyl-2-**[^18^ **F**]**fluoropropionate**	200 min	—	[39]
	*Disulfide bridge	via amide formation			

**α**_**v**_ **β**_6_ **targeting**					

[^18^F]FBA-A20FMDV2	**NAVPNLRGDLQVLAQKVART**	**solid-phase p-**[^18^ **F**]**fluorobenzoyl labeling**	130 min	3.6% (dc)	[23]
	(derived from foot-and-mouth disease virus)	via amide formation		37 GBq/µmol	

[^18^F]FBA-(PEG_28_)_2_-	**NAVPNLRGDLQVLAQKVART**	**solid-phase p-**[^18^ **F**]**fluorobenzoyl labeling**	—	—	[40]
A20FMDV2	PEG linker	via amide formation			

[^18^F]FBA-C_6_-ADIBON_3_-PEG_7_- A20FMDV2	**NAVPNLRGDLQVLAQKVART**	[^18^ **F**]FBA-C _6_ **-ABIO**	45 min	12% (dc) 70 GBq/µmol	[41]
N-terminal azido-PEG derivatized	(^18^F-labelled cyclooctyne derivative) Cu-free strain-promoted “click chemistry”		

[^111^In]DTPA-A20FMDV2	**NK(biotinyl)VPNLRGDLQVLAQKVART**	^ 111^ **In** via complexation	—	—	[42]
	N-terminal DTPA conjugated 2nd amino acid replaced by biotinyl-lysine			

[^64^Cu]CB-TE1A1P-PEG_28_- A20FMDV2	**NAVPNLRGDLQVLAQKVART**	^ 64^ **Cu** via complexation	15 min^2^	>98% (ns) 22 GBq/µmol	[43]
N-terminal tetraazabicyclo[6.6.2.]hexadecane derivative conjugated		

[^64^Cu]DOTA-S_0_2	**RSLARTDLDHLRGR**	^ 64^ **Cu** via complexation	—	80% (ns) 18.5 GBq/µmol	[44]
	(sequence engrafted into loop 1 of a acyclized cystine knot scaffold) Loop 2 serine-rich N-terminal DOTA conjugation		

^ 18^F-fluorobenzoate-R_0_1	**ILNMRTDLGTLLFR**	**succinimidyl-p-**[^18^ **F**]**fluorobenzoate** via amide formation at N-terminus	45 min^2^	7% (dc) —	[24]
	(sequence engrafted into loop 1 of a acyclized cystine knot scaffold) Loop 2 arginine-rich		

[^99m^Tc]SAAC-S_0_2	**RSLARTDLDHLRGR**	**[** ^ 99m^ **Tc(H**_ 2_ **O)**_ 3_ **(CO)**_ 3_ **]** ^−^ Tc-tricarbonyl method	60 min^2^	40% (ns) 15 GBq/µmol	[45]
	(sequence engrafted into loop 1 of a acyclized cystine knot scaffold) Loop 2 serine-rich N-terminal SAAC^6^ modified		

^1^SAA: galactose based sugar amino acid.

^2^Synthesis time only (overall production time depends on several parameters, e.g., type of automated system, labeling technique, and postprocessing).

^3^TRAP: 1,4,7-triazacyclononane-1,4,7-tris(2-carboxyethyl)methylenephosphinic acid.

^4^Pra: propargyl glycine.

^5^Precursor is produced via isotopic exchange.

^6^SAAC: single amino acid chelate.

^7^dc: decay corrected.

^8^ns: not specified.
